# Effects of an oil spill in a harbor assessed using biomarkers of exposure in eelpout

**DOI:** 10.1007/s11356-014-2890-z

**Published:** 2014-05-13

**Authors:** Joachim Sturve, Lennart Balk, Birgitta Liewenborg, Margaretha Adolfsson-Erici, Lars Förlin, Bethanie Carney Almroth

**Affiliations:** 1 Department of Biological and Environmental Sciences, University of Gothenburg, Box 463, SE-405 30 Göteborg, Sweden; 2 ITM, Department of Applied Environmental Science, Stockholm University, SE-106 91 Stockholm, Sweden

**Keywords:** Oil spill, Polycyclic aromatic hydrocarbons, Eelpout, Biomarkers, EROD, DNA adducts

## Abstract

Oil spills occur commonly, and chemical compounds originating from oil spills are widespread in the aquatic environment. In order to monitor effects of a bunker oil spill on the aquatic environment, biomarker responses were measured in eelpout (*Zoarces viviparus*) sampled along a gradient in Göteborg harbor where the oil spill occurred and at a reference site, 2 weeks after the oil spill. Eelpout were also exposed to the bunker oil in a laboratory study to validate field data. The results show that eelpout from the Göteborg harbor are influenced by contaminants, especially polycyclic aromatic hydrocarbons (PAHs), also during “normal” conditions. The bunker oil spill strongly enhanced the biomarker responses. Results show elevated ethoxyresorufin-*O*-deethylase (EROD) activities in all exposed sites, but, closest to the oil spill, the EROD activity was partly inhibited, possibly by PAHs. Elevated DNA adduct levels were also observed after the bunker oil spill. Chemical analyses of bile revealed high concentrations of PAH metabolites in the eelpout exposed to the oil, and the same PAH metabolite profile was evident both in eelpout sampled in the harbor and in the eelpout exposed to the bunker oil in the laboratory study.

## Introduction

Oil spills occur commonly, and chemical compounds originating from oil spills, such as polycyclic aromatic hydrocarbons (PAHs), are widespread in the aquatic environment (Beyer et al. 2010; de Hoop et al. 2011). Aquatic organisms protect themselves against the harmful effects of exposure to these and other xenobiotics via molecular and cellular defense systems, such as detoxifying enzymes and molecules, metal-binding proteins, and trapping of foreign toxic compounds by lysosomes. These responses, as well as any cellular or molecular damage that may occur as a result of exposure, are often used in monitoring and assessment programs addressing the environmental impact of pollutants (van der Oost et al. 2003). Interactions between pollutants and biochemical and physiological functions in fish, detected as subcellular, cellular, or organ disturbances, are referred to as biomarkers and can serve as early warning signs, indicating possible disturbances in reproduction success, growth, or survival of the fish (Forlin et al. 1986; Haux and Forlin 1988).

Many of the compounds found in oil, for example PAHs, are oxidatively metabolized in fish by detoxifying enzymes in the phase I cytochrome P450 system, also called the CYP system. The CYP1A subfamily belongs to the superfamily of CYPs, and is important in the phase I detoxification reactions in fish. The phase I metabolites (e.g., hydroxylated PAHs) are further metabolized by phase II systems to form water-soluble conjugates that are excreted into the bile (Leonard and Hellou 2001). The metabolites can be detected by high-performance liquid chromatography (HPLC)/fluorescence or by gas chromatography/mass spectrometry (GC/MS) after hydrolysis of the conjugates. The bioconcentration factor bile/water can be up to 10^6^ in fish, which means that low concentrations of a contaminant in the aquatic environment can be detected via metabolites in the bile. Identification of PAH metabolites in the bile can be used to distinguish between petrogenic and pyrogenic PAH exposure. Petroleum PAH are often dominated by alkylated two- and three-ringed aromatics, while pyrogenic PAH are dominated by four- and five-ringed aromatics (Anderson and Lee 2006). Many PAHs, or their metabolites, are known to be toxic and/or carcinogenic (Aas et al. 2001), and PAHs are considered the most toxic of all petroleum compounds (Yanik et al. 2003). The induction of CYP1A in fish, usually measured as increased activity of ethoxyresorufin-*O*-deethylase (EROD), is considered to be the most responsive and consistent biomarker for aryl hydrocarbon (AH)–receptor ligands, such as PAHs and planar PCBs (Goksøyr and Förlin 1992). However, the relationship between exposure to PAHs and consequent induction of EROD activity is not always positively correlated. High levels of PAHs have been shown in some cases to inhibit EROD activity (Schiedek et al. 2006). PAHs are also known to be genotoxic, and they can be bioactivated via the cytochrome P450 system, resulting in more toxic intermediate metabolites (Baird et al. 2005). These metabolites are toxic and effects include DNA adducts which can be quantified (Lyons et al. 2004; Amat et al. 2006; Malmström et al. 2009). PAHs are hydrophobic and semivolatile, factors that contribute to their accumulation in sediments and biological tissues and to their persistency in the environment (Yanik et al. 2003), and the major degradation pathways, which are microbial, are highly dependent upon environmental conditions (Haritash and Kaushik 2009).

Previous studies have addressed the effects of an oil spill on eelpout (Frenzilli et al. 2004; Carney Almroth et al. 2005), and have shown oxidative stress and DNA damage as a result. The eelpout (*Zoarces viviparus*) is a viviparous species of blenny that is commonly used in European environmental biomonitoring campaigns due to the fact that its range reaches from Norway to northern France including also the Baltic Sea (Whitehead et al. 1984). Several additional characteristics of this species make it suitable for field studies, e.g., the fish are relatively stationary, making it possible to correlate exposure conditions to physiological responses in wild fish, and the viviparous nature of their reproduction allows for studies addressing reproduction and maternal effects. This species has been used in Swedish biomonitoring programs for more than 20 years as a sentinel species for environmental pollution (Vetemaa et al. 1997; Ronisz et al. 1998; Larsson et al. 2000; Frenzilli et al. 2004; Carney Almroth et al. 2005; Ronisz et al. 2005; Sturve et al. 2005; Gercken et al. 2006), resulting in considerable amounts of data concerning its basic physiology, responses to pollutant exposure, and consistent information describing the reference sites.

In this study, we have addressed the effects of exposure to bunker oil on feral eelpout. In June 2003, between 10 and 100 t of bunker oil containing around 25 % PAHs were accidentally released from a storage facility in Göteborg harbor in Sweden and spread into the outer parts of the Gothenburg archipelago. Eelpout were sampled before, and 2 weeks and 5 months after the oil spill and levels of CYP1A protein, hepatic EROD activity and DNA adducts were measured. We also measured the amounts of PAH metabolites in bile as their hydroxylated transformation products as well as bile fluorescent aromatic compounds (FAC), and verified their use as biomarkers of exposure.

## Material and methods

### Chemicals

Sodium acetate, acetic acid, and potassium carbonate, all of analytical grade (Merck, Darmstadt, Germany), *n*-hexane “LiChrosolve” (Merck, Darmstadt, Germany), acetic anhydride of analytical grade (Riedel-de Haen, Seelze, Germany), and β-glucuronidase (102 100 units/mL of β-glucuronidase and 290 units/mL of sulfatase) (Sigma-Aldrich Chemie Gmbh, Steinheim, Germany), methyl-*t*-butyl ether of HPLC grade (Rathburn Chemicals Ltd, Walkerburn, UK). Analytical standards 2-OH-naphthalene, 1-OH-fluorene, 1-OH-phenanthrene, 1-OH-pyrene (Sigma-Aldrich Chemie Gmbh, Steinheim, Germany), and β,β′-binaphthyl were purchased from Larodan Fine Chemicals AB, Malmö, Sweden. Ethanol, acetone, and peanut oil were all of analytical grade.

### Field samplings

**Fig. 1:**
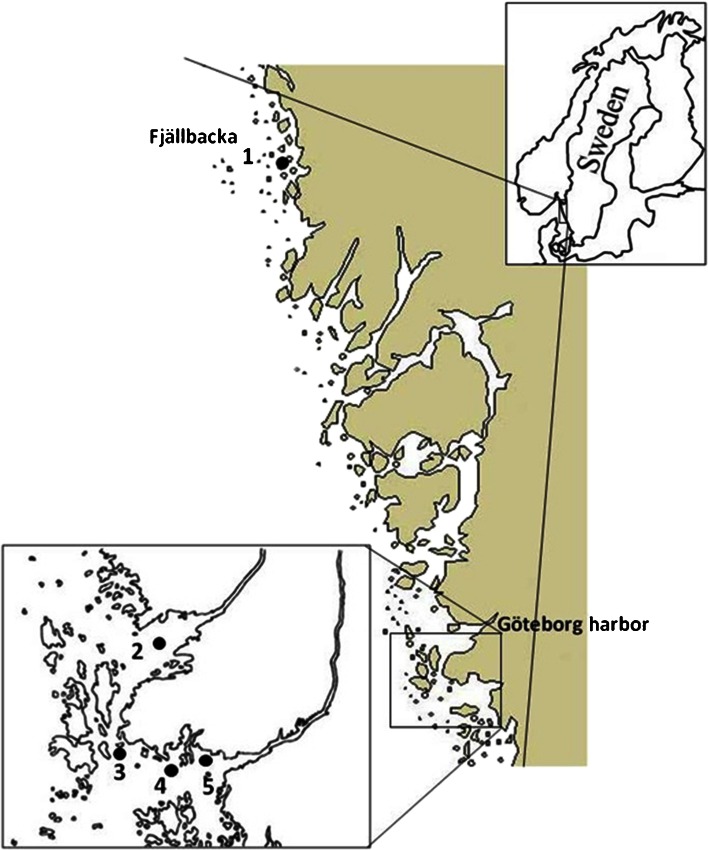
Map of field sampling sites, showing two reference sites (*1* Fjällbacka, national reference site; *2* Nordre Älv, local reference site) and three sites within the harbor (*3* Hjuvik, *4* Skalkorgarna, *5* Aspholmarna).

Eelpout were captured with fyke nets by local fishermen at four sites around the River Göta älv estuary, Göteborg. The sites were Nordre älv, a local reference site; Hjuvik in the outer harbor; Skalkorgarna, the site located within the oil harbor; and the inner harbor site, Aspholmarna. Fish were also taken from the national reference site, Fjällbacka, 150 km north of the River Göta älv estuary. Figure 1 shows a map displaying the sampling sites. Fish were captured at three time points. The first sampling was prior to the spill in May when samples were taken as part of a regional biomonitoring campaign. Biomarker results from this sampling campaign have already been presented in Sturve et al. (2005). Samples were also collected in July (2 weeks after the oil spill) and in November, approximately 5 months after the oil spill. In July, sampling had to be repeated after 4–5 days at the Nordre älv and Aspholmarna sites due to low catches at the first fishing time point. Due to practical reasons, it was not possible to take samples from the national reference site, Fjällbacka, in July.

### Laboratory studies

Two laboratory studies were performed with bunker oil from the batch that was spilled into the harbor. The oil was a gift from the Västra Götaland County administration. Eelpout for the laboratory studies were collected from Grundsund, Sweden, with the help of local fishermen. This site is located 65 km north of Göteborg city and is considered to be an unpolluted site. The fish were transported to the Department of Biological and Environmental Sciences, Gothenburg University, and kept in aerated and filtered sea water (32 ‰) at 12 °C and 12:12 light cycle for up to 2 weeks for acclimatization.

In the first exposure study, eelpout was exposed to the oil through water exposure for 96 h. Female eelpout (*n* = 36) were divided into four 600-L tanks (giving 1 g of fish per liter of water) with aerated seawater at approximately 12 °C and exposed to the oil in a static system. Besides the control group, the fish were exposed to three different doses of the oil (10, 100, and 1,000 μg/L), dissolved in 60 mL of acetone. The control group was exposed to 60 mL of acetone alone.

The second exposure study aimed to study DNA adducts formation and was therefore longer. Ten female eelpout were injected intraperitoneally (i.p.) with 100 mg bunker oil/kg fish. After 10 days, five of the fish were re-injected with the same, resulting in two exposure groups (five fish per group), injected once (Inj 1) and injected twice (Inj 2). The control group (five fish) was injected with the carrier alone (peanut oil). All of the fish were sampled after 21 days. Five fish were sampled immediately when arriving to the laboratory, thus forming an extra control group (0 sample).

### Sampling

Eelpout were killed by a sharp blow to the head and the weight and length was recorded. The fish were cut open, bile was collected with a syringe, and the liver excised, weighed, and frozen in liquid nitrogen. Before freezing, the liver was divided into two pieces; one for enzymatic analysis and one for DNA adduct analysis. Bile samples from female fish were pooled (five fish per pool) in order to obtain sufficient volumes. The pooled bile samples were put in dark glass bottles, frozen on dry ice, and kept in −20 °C until analysis.

### Biochemical analysis

The microsomal fraction was obtained following the protocol described by Forlin et al. (1984). Livers were homogenized using glass/Teflon® for 3 × 5 s in four volumes of homogenization buffer (0.1 M Na^+^/K^+^-phosphate buffer (pH 7.4) containing 0.15 M KCl). The homogenate was centrifuged in two steps, first 10,000 × *g* for 20 min at 4 °C followed by 105,000 × *g* for 60 min at 4 °C. The supernatant (cytosol fraction) was collected and the pellet (microsomal fraction) resuspended in homogenization buffer containing 20 % glycerol and stored at −80 °C until analysis.

*EROD activity* was measured in the microsomal fraction according to a spectrofluorometric method described by Forlin et al. (1984) using rhodamine as standard.

*CYP1A levels* were determined in the microsomal fraction with an enzyme-linked immunosorbent assay (ELISA) according to Ronisz and Förlin (1998). Due to the lack of standard CYP1A protein, the results are presented as absorbance and not as absolute CYP1A levels, giving relative differences between groups.

*Protein levels* in the microsomal fractions were determined according to the method described by Lowry et al. (1951) using bovine serum albumin as standard.

### DNA adduct measurement

Deep-frozen liver tissue from blenny were semithawed, and the DNA extracted and purified according to previous reports (Dunn et al. 1987; Reichert and French 1994; Ericson and Balk 2000) and slightly modified as described previously (Ericson et al. 1998; 2000). DNA adducts were enriched using the Nuclease P1 method, 0.8 μg nuclease P1/μg DNA, and a 45 min incubation period (Reddy and Randerath 1986; Beach and Gupta 1992). Finally, the DNA adducts were radiolabelled using 5′-[γ-^32^P]triphosphate([γ-^32^P]ATP) and T_4_ polynucleotide kinase (Aas et al. 2000). Separation and clean-up of adducts was performed by multidirectional thin layer chromatography (TLC) on laboratory produced polyethyleneimine cellulose sheets, described as suitable for adducts formed from large hydrophobic xenobiotics, such as four to six ring, PAHs (Reichert and French 1994; Ericson et al. 1998, 1999).

In addition, several quality control experiments were performed in parallel to the analysis of the eelpout samples. All these quality assurance experiments strongly suggested a faultless assay for the DNA adduct measurements.

### Bile fluorescent aromatic compounds

Synchronous fluorescence spectrometry of PAH metabolites in bile was performed following the protocol described by Aas et al. (2000). Individual bile samples were diluted in 1:800 in 48 % ethanol with further dilution (1:1,600, 1:3,200, 1:6,400, or 1:12,800) when necessary and submitted to synchronous fluorescence scan with Δλ = 42 nm. The peak corresponding to pyrene-like compounds (emission wavelength 383) was quantified by integrating and calculating the peak area between the emission wavelengths 365 to 400. PAH metabolite levels are expressed as arbitrary fluorescence.

### Speciation of PAH metabolites in bile

Approximately 200 mg bile was enzymatically hydrolyzed by adding 20 μL β-glucuronidase (102,100 units/mL) in 1 mL 0.2 M acetic acid buffer, pH 5, and incubated for 2 h at 37 °C. Free compounds were extracted with 2 × 3 mL hexane/methyl-*t*-butyl-ether (1:1) after addition of 2 mL water and 0.5 g NaCl. The combined organic phases were evaporated to dryness; the residue was dissolved in 2 mL hexane. For acetylation of the hydroxyl groups, 100 μL acetic acid anhydride/pyridine (1:1) was added, and the mixture was heated at 60 °C for 30 min. The combined organic phases were concentrated and analyzed by GC/MS-selected ion monitoring after addition of internal standard (100 μL β,β′-binaphthyl). For quantitation purposes, the following ions were used: 115, 144 (2-OH-naphthalene), 165, 194 (1-OH-phenanthrene), 189, 218 (1-OH-pyrene), and 182 (2-OH-fluorene). The acetylated extracts were also analyzed by full-scan gas chromatography/mass spectrometry for identification of other hydroxylated compounds. By interpretation of mass spectra, and by comparing retention times in the GC/MS chromatogram, 10 methylated hydroxylated PAH metabolites were tentatively identified. An estimation of their concentrations were made using the following ions: 158, 200 (M^+^) for CH_3_-OH-naphthalenes, 172, 214 (M^+^) for (CH_3_)_2_-OH-naphthalenes, 186, 228 (M^+^) for (CH_3_)_3_-OH-naphthalenes, 196, 238 (M^+^) for CH_3_-OH-fluorenes, 210,252 (M^+^) for (CH_3_)_2_-OH-fluorenes, 208, 250 (M^+^) for CH_3_-OH-phenanthrenes, 222, 264 (M^+^) for (CH_3_)_2_-OH-phenanthrenes, 232, 274 (M^+^) for CH_3_-OH-pyrenes, and 246, 288 (M^+^) for (CH_3_)_2_-OH-pyrenes. The extracts were analyzed in splitless mode on a Hewlett-Packard 5890 series II GC (Avondale, PA, USA) coupled with a JEOL low-resolution automass with electron ionization, 70 eV (Stoughton, WI, USA). The ion source was 200 °C, and the interface was 250 °C. Helium was used as carrier gas. The column (30 m × 0.25 mm MSDB5 with a phase thickness of 0.25 μm, J&W Scientific, Folsom, CA, USA,) was held at 90 °C for 1 min, then quickly raised to 200 °C followed by an increase of 10 °C/min up to 300 °C. The injector temperature was 275 °C. Two pools per site were analyzed for the field samples while only one pool per exposure condition was analyzed in the laboratory exposure study. For the Nordre älv and Aspholmarna sites, one pool per sampling time point was selected for analysis.

### Statistical analyses

Data was analyzed with one-way analysis of variance (ANOVA) followed by Student–Newman–Keuls test using the software SPSS® version 18 for Windows. Data not displaying homogeneity of variance (Leven’s test) were log transformed prior to testing. Data are presented as mean ± standard error (SEM), and the significance level was set at *p* < 0.05.

## Results

### Laboratory studies

#### EROD activity

**Fig. 2:**
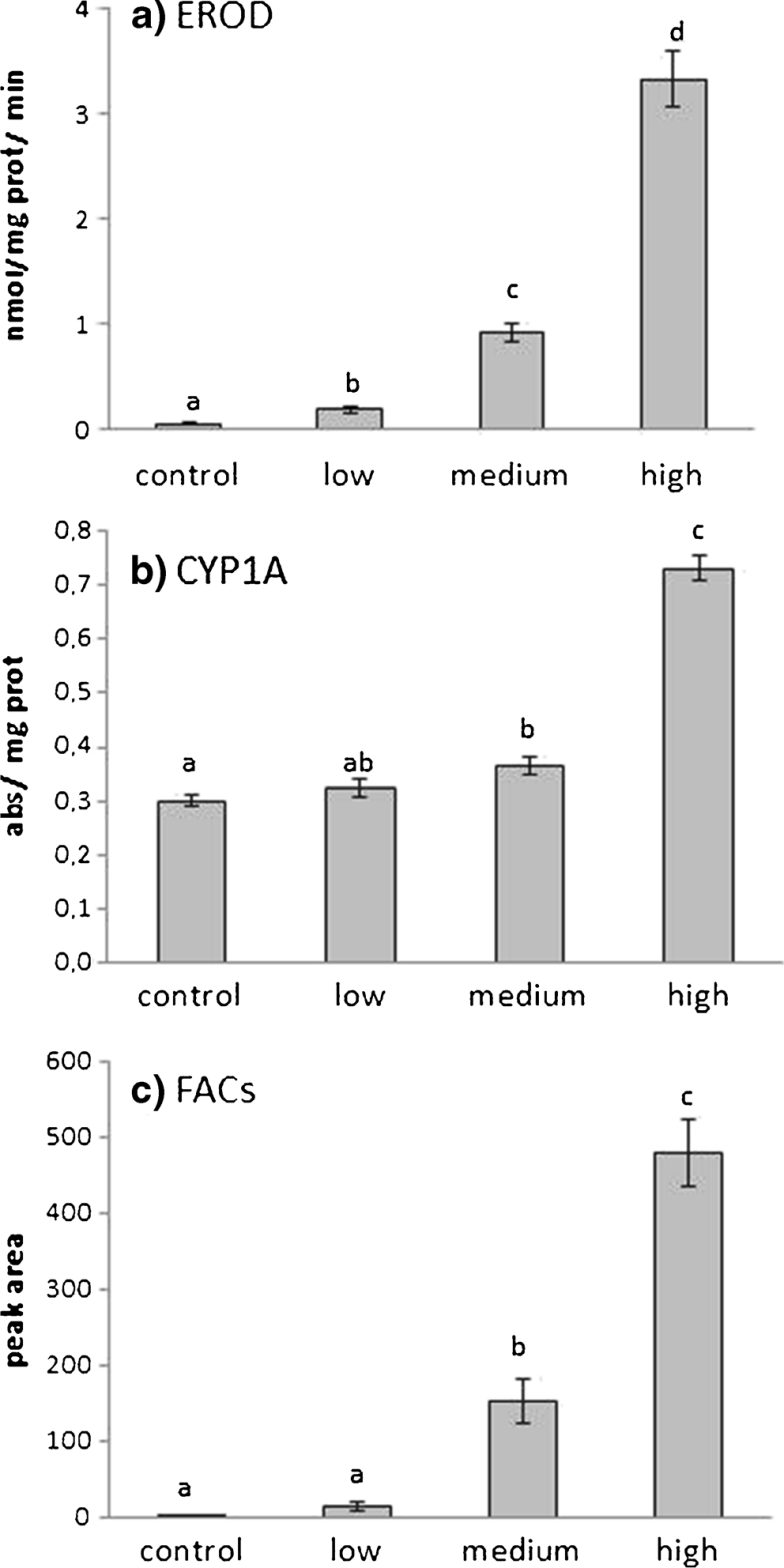
Results from the 4-day laboratory water exposure study using bunker oil. EROD activities (**a**), levels of CYP1A protein (**b**), and PAH metabolites (FACs) in bile (**c**). Fish were exposed in four groups, control, low dose (10 μg/L), medium dose (100 μg/L), and high dose (1,000 μg/L). Results are shown as mean ± standard error, *n* = 8. *Letters* (*a*, *b*, *c*) indicate statistical differences, *p* < 0.05

EROD activities showed a dose-dependent elevation after exposure to the crude oil in the laboratory, all doses resulting in significantly elevated EROD activities compared to the control. The low dose resulted in 3 times elevation, the middle dose 18 times elevation, and the high dose 72 times elevation in EROD activity (Fig. 2a).

#### CYP1A levels

Hepatic CYP1A levels in eelpout were significantly elevated in the middle and the high dose compared to the control group after oil exposure in the laboratory (Fig. 2b).

#### DNA adduct levels

DNA adduct levels in the livers were analyzed in eelpout exposed to the oil for 3 weeks through IP injections. Eelpout from both control groups, 0 sample and carrier control, were found to have similar levels of DNA adducts (2.6 ± 1.1 and 2.9 ± 0.8, respectively). Also, eelpout receiving a single or two doses of bunker oil were found to have similar levels of DNA adducts (16.4 ± 9.3 and 15.2 ± 4.9, respectively). Both groups exposed to bunker oil showed statistically significant elevation in DNA adduct levels compared to both control groups (data not shown). Units for the DNA adducts are nmol adducts/mol normal nucleotides.

#### FACs

FAC levels in bile of eelpout exposed to oil showed similar dose-dependent pattern as EROD activity. The middle and high exposures resulted in significantly elevated levels of FAC metabolites in bile compared to the control (Fig. 2c).

#### Hydroxylated PAH metabolites

The concentration of hydroxylated PAH metabolites was analyzed in bile from the laboratory exposure, and the results are displayed in Table 1. After the 3-week long exposure study via i.p. injection, the total amount was approximately 35 times higher in the exposed fish compared to the control. Results from the water exposure study show a dose–response relationship with approximately 8 times higher levels of hydroxylated PAH metabolites in the fish exposed to the low dose, approximately 97 times higher in the middle dose and approximately 600 times higher in the high dose.

**Table 1:** Amount of hydroxylated naphthalenes, fluorenes, phenanthrenes, and pyrenes in bile from eelpout exposed to the bunker oil in a laboratory study. Eelpout were exposed to for 96 h to three oil concentrations (10, 100, and 1,000 μg/L) through water exposure. Levels are shown as nanogram PAH per gram of bile. The total amount of the analyzed PAH and their different grades of methylation are shown in the column. Identification of methylated PAH is based on interpretation of mass spectra and the amount is approximated by assuming the same response factors as for the unmethylated congener

Exposure	OH naphthalenes				OH fluorenes			OH phenanthrenes			OH pyrenes			Total
	C0	C1^a^	C2^a^	C3^a^	C0	C1^a^	C2^a^	C0	C1^a^	C2^a^	C0	C1^a^	C2^a^	
	ng/g f.w	ng/g f.w	ng/g f.w	ng/g f.w	ng/g f.w	ng/g f.w	ng/g f.w	ng/g f.w	ng/g f.w	ng/g f.w	ng/g f.w	ng/g f.w	ng/g f.w	
Control	<0.5	<0.05	<0.5	<0.5	<1	<0.5	<0.5	2	<5	<5	100	<5	<10	102
Low dose	<0.5	<0.05	8	2	4	3	3	15	200	13	300	230	30	809
Middle dose	0.8	20	160	25	67	51	46	480	1,300	290	3,500	3,500	300	9,740
High dose	3.5	200	1,400	230	440	410	320	3,100	7,800	2,100	>23,000	>20,000	990	>60,000

^a^Values are based on interpretation of mass spectra

### Field study

#### EROD activity

**Fig. 3:**
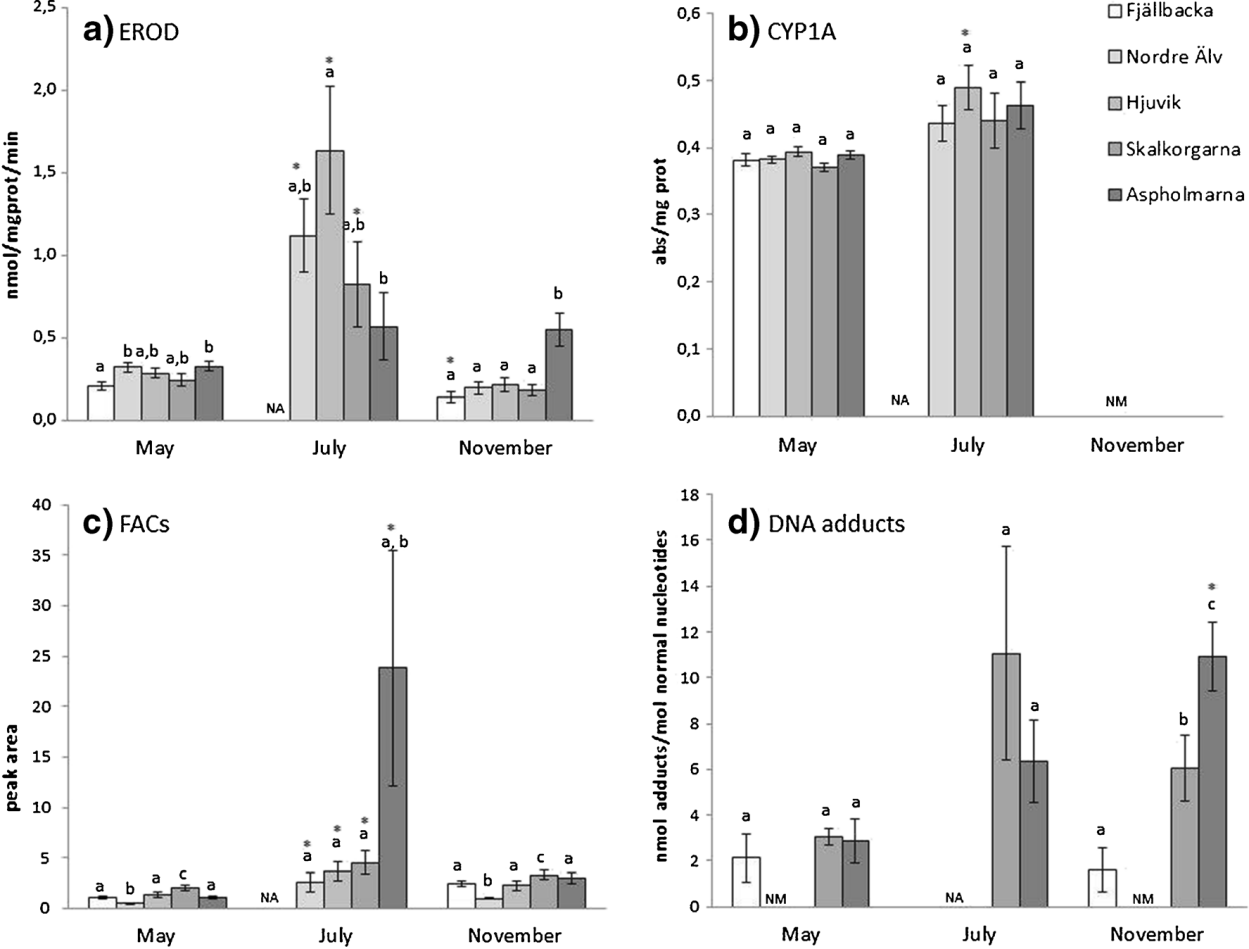
Effects of oils spill on female eelpout. EROD activities (**a**), levels of CYP1A protein (**b**), PAH metabolites (FACs) in bile (**c**), and levels of DNA adducts (**d**) are shown. Results are shown as mean ± standard error. *Letters* (*a*, *b*, *c*) indicate statistical differences between sites at one time point. *Asterisk* indicates significant difference in levels at a certain site during or after the oils spill, compared to before (May), *p* < 0.05. *NA* not available, *NM* not measured

EROD activities showed significant differences between the control sites Fjällbacka and Nordre älv and Aspholmarna, within the harbor, in the May sampling (Fig. 3a). In July, the EROD activities were significantly higher in the Nordre älv, Hjuvik, and Skalkorgarna sites compared to the same sites at the May sampling. Hjuvik showed significantly higher EROD activities compared to the inner harbor site, Aspholmarna (Fig. 3a). In November, fish from Aspholmarna showed significantly higher levels of EROD that the four other sites in the River Göta älv estuary and the national reference site, Fjällbacka.

#### CYP1A levels

Hepatic CYP1A levels were significantly higher in eelpout caught at Hjuvik following the oil spill in July when compared to fish from the same site caught prior to the spill in May. CYP1A levels did not differ significantly between sites in May or in July (Fig. 3b).

#### DNA adduct levels

DNA adduct levels did not differ between the sites in May (Fig. 3d). In July, the DNA adduct levels were highest at the Skalkorgarna site; however, the difference was not statistically significant between sites or compared to May. In November, the Skalkorgarna site showed a significantly higher DNA adduct levels compared to Fjällbacka; the Aspholmarna site also displayed significantly higher DNA adduct levels compared to Fjällbacka, and levels in Aspholmarna were significantly higher than both of the other sites.

**Fig. 4:**
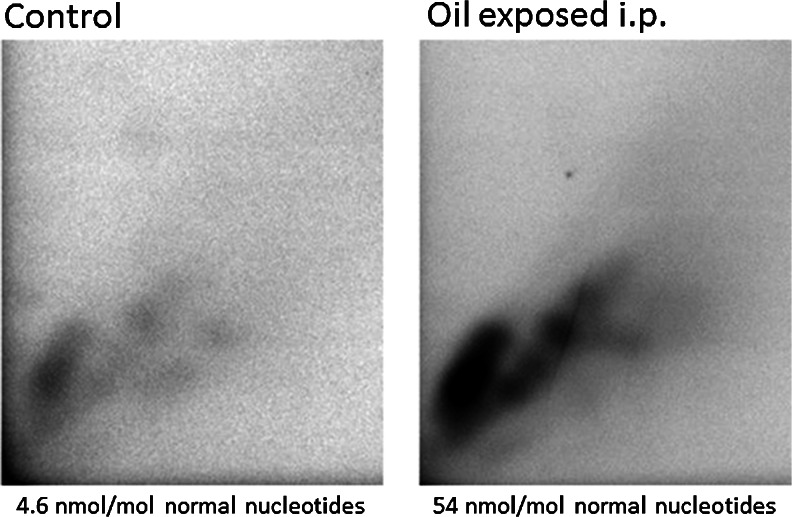
Autoradiogram images showing DNA adduct patterns in wild caught eelpout used in a laboratory exposure study. Example of one control fish and one fish exposed to the oil through i.p. injection. Similarities in pattern suggest that the eelpout had been exposed to low levels of the oil prior to capture

A comparison of the autoradiogram fingerprints from the laboratory exposure control fish (0 sample from the reference site Grundsund) and the fish-exposed i.p. for bunker oil at the laboratory show several similarities (Fig. 4).

#### FACs

Result from FACs measurements showed that eelpout captured at the Aspholmarna site in May had significantly higher FACs in the bile compare to the other sites, while the lowest levels were measured in fish from Nordre älv. All sites had significantly higher FAC levels in July compared to the same sites in May (Fig. 3c), and levels at Aspholmarna were highest. Four months after the spill, the amounts of FACs were similar to those present prior to the spill.

#### Hydroxylated PAH metabolites

Results from the chemical analysis of hydroxylated naphthalenes, pyrenes, fluorenes, and phenanthrenes of different degree of methylation in bile from eelpout captured at the sampling sites after the oil spill (July) are presented in Table 2. The sum of identified chemicals show that eelpout from the Aspholmarna site contained most hydroxylated PAHs followed by the Skalkorgarna, Hjuvik, and finally Nordre älv sites with the lowest levels. However, bile from fish caught in Nordre älv contained approximately 20 times higher PAH metabolites compared to bile from the control fish used in the laboratory exposure studies (Tables 1 and 2). Results from the sites Hjuvik and Skalkorgarna, where two pools obtained at the same sampling time point were analyzed, show similar results. The repeatability in these results demonstrates robustness of our method. In the Nordre älv and Aspholmarna sites, results show higher levels in the fish captures in the first sampling time point compared to the second (Table 2).

**Table 2:** Amount of hydroxylated naphthalenes, fluorenes, phenanthrenes, and pyrenes in bile from eelpout captured at different locations (see Fig. 1 for map) approximately 2 weeks after the oil spill. Levels are shown as nanogram PAH per gram of bile. The total amount of the analyzed PAH and their different grades of methylation are shown in the column. Identification of methylated PAH is based on interpretation of mass spectra and the amount is approximated by assuming the same response factors as for the unmethylated congener

Exposure	Date	OH naphthalenes				OH fluorenes			OH phenanthrenes			OH pyrenes			Total
		C0	C1^a^	C2^a^	C3^a^	C0	C1^a^	C2^a^	C0	C1^a^	C2^a^	C0	C1^a^	C2^a^	
		ng/g f.w	ng/g f.w	ng/g f.w	ng/g f.w	ng/g f.w	ng/g f.w	ng/g f.w	ng/g f.w	ng/g f.w	ng/g f.w	ng/g f.w	ng/g f.w	ng/g f.w	ng/g f.w
Blank		<0.5	<0.05	<0.5	<0.5	<1	<0.5	<0.5	<5	<5	<5	<5	<5	<10	<10
Nordre älv1	4/7	2.6	25	66	7	24	11	10	114	180	41	620	230	<10	1,330
Nordre älv2	10/7	2.0	16	29	5	12	8	5	19	79	12	310	52	<10	549
Hjuvik1	3/7	1.8	17	100	7	40	14	<0.5	140	200	20	960	600	<10	2,100
Hjuvik2	3/7	1.9	28	86	6	29	12	12	120	280	14	850	330	<10	1,769
Skalkorgarna1	4/7	2.0	18	62	5	30	17	15	100	470	28	1,100	340	<10	2,187
Skalkorgarna2	4/7	2.0	19	62	5	25	11	10	110	200	26	730	300	<10	1,500
Aspholmarna1	30/6	13	130	790	110	240	230	100	2,400	4,100	1,100	9,600	4,900	260	23,973
Aspholmarna2	4/7	4.8	190	540	24	63	13	23	250	330	82	1,400	860	99	3,879

^a^Values are based on interpretation of mass spectra

### Correlations

Correlations between data obtained from the field and laboratory exposure studies were calculated. In the field study, EROD activity correlated positively to DNA adducts (*p* = 0.006). Also, CYP1A levels correlated strongly to EROD activity. However, we noted an outlier in the data; samples from Aspholmarna collected in July, 2 weeks after the oil spill, displayed lower EROD activities than would be predicted by the correlations to CYP1A (Fig. 3a, b). In addition, when EROD results from the Aspholmarna site in July were removed from the analysis, EROD activity also correlated to the sum of PAHs in bile (*p* < 0.001). Also, a comparison of total PAHs measured by FACs and the sum of the PAHs measured by chemical analysis revealed a strong correlation (*p* = 0.008).

Following the exposure to oil in the laboratory, results showed strong correlations between EROD and CYP1A protein levels and between EROD and total PAHs measured by FACs in bile. There was also a significant correlation between the two methods of measuring PAHs (FACS and HPLC measurements) in the fish from the field study (*p* = 0.044).

The pattern of alkylated PAH metabolites in the field samples and the samples from the laboratory study were almost identical (see Tables 1 and 2).

## Discussion

PAHs are one of the most important groups of organic contaminants found in the aquatic environment, and one source of this input into waters is from oils spills which unfortunately occur commonly (petrogenic sources). Another source is incomplete combustion of organic matter (pyrogenic sources). PAHs are genotoxic, and their metabolism results in intermediate compounds that can bind directly to DNA, RNA, lipids and proteins, or mediate their toxic effects through the production of reactive oxygen species. The oil spill that occurred in Göteborg harbor provided an opportunity to investigate the effects of exposure to extremely high levels of PAHs in eelpout in the natural environment, and to address the usefulness of different methods to assess these effects.

The PAHs originating from petrogenic sources are often dominated by two- and three-ringed aromatics, while PAH from pyrogenic sources are dominated by four- and five-ringed aromatics (Anderson and Lee 2006). The ratio of the sum of methylphenanthrene to phenanthrene (MP/P ratio) are used to distinguish between petrogenic (MP/P >2) and pyrogenic PAH (MP/P <0.5) (Boonyatumanond et al 2006). The MP/P ratios for the different field sampling sites ranged from 1.5 to 2.2 indicating that the PAH originated from petrogenic sources. Also, the pattern of alkylated PAH metabolites in the bile of fish caught in the recipient of Göteborg harbor and in the bile of fish exposed in the laboratory to the bunker oil that was spilled in the harbor was almost identical in most cases. This is evidence that that the wild fish were actually exposed, and accumulated substances from the spill, even though anecdotal evidence suggested that the oil was insoluble and formed agglomerates that could easily be removed from the water (personal conversation with city representatives). The amounts of specific PAH metabolites present in bile were measured using GC/MS, and these results were compared to measurements conducted using the FACs which indicates the sum of total PAH metabolites. Synchronous fluorescence spectrometry of PAH metabolites (FAC) is a sum parameter that measures pyrene-like compounds. This means that you also measure fluorescing compounds other than PAH metabolites that are present in the oil. It is a rapid and versatile method to compare the amount of pyrene-like compounds in fish from different sites and within groups. The results from FACs are not directly comparable to the GC/MS method, which determines the amount of specific isomers of PAH metabolites after enzymatic hydrolysis of glucuronides. However, results from both methods correlated well, indicating that the more simple FACs method is suitable to assess exposure to PAHs. If there is need for more specific analysis, e.g., to distinguish between sources of pollution, the GC/MS method is preferable. Results from the Nordre älv and Aspholamarna sites, where fish were captured at two different time points, show that the levels of specific PAH metabolites in the bile decreased rapidly. Fish were apparently able to metabolize and excrete a large portion of the PAHs within a few days.

Exposure to the oil spill in the harbor resulted in significantly elevated levels of CYP1A proteins and to hepatic EROD activity. Fish captured at all sites in the harbor also had elevated levels of PAH metabolites in their bile in July, shortly after the spill. PAHs are known to bind to the AH receptor which plays an important role in the induction of CYP1A proteins (Schlenk et al. 2008). In November, EROD activities and PAH metabolites were at levels similar to those measured in samples taken prior to the spill, indicating that PAH load in the body had decreased. Several studies have addressed the seasonal variations of EROD activity in fish (Ronisz et al. 1999; Rotchell et al. 1999), indicating that highest levels are normally recorded in the late winter months of February and March. In the current study, levels were lowest during the spring and fall samplings. The fact that the oil spill occurred in the end of June, and samples were collected in July, when EROD levels would normally be at their lowest, is another indication of the extreme induction of this enzyme activity in the exposed fish.

DNA adducts were also measured in selected samples, and results showed that levels tended to increase following the oil spill, but the increase was not significant in July due to high variation. However, DNA adducts were present in the harbor at significantly higher levels several months after the spill indicating more long-term chronic effects, a result of interactions between PAH metabolites and DNA (Aas et al. 2000). This relationship between PAH exposure, acute induction of EROD, followed by DNA adduct formation after chronic exposure, has been demonstrated in a laboratory exposure study using flounder exposed via food (Reynolds et al. 2003).

Our results are in agreement with other studies which have indicated that biomarkers such as EROD activity or PAH in bile are useful to investigate short-term or acute effects of exposures (French et al. 1996; Aas et al. 2000). DNA adducts may be more useful as biomarkers in chronic effect situations, and have been shown to occur in feral fish (Wirgin and Waldman 1998) and to increase linearly with exposure to increasing concentrations of PAHs and with time (French et al. 1996). Our laboratory exposure also indicated that EROD activity and CYP1A levels were induced following an acute 96-h water exposure study, and that PAH metabolites can be found in bile at this time as well. There was a significant increase in DNA adducts 21 days following a single i.p. injection of the crude oil.

The laboratory study was conducted in order to confirm results seen in the field and to provide controlled samples to be used in assessment of DNA adducts. Exposure to the bunker oil via water resulted in uptake of PAHs, which is evident in the PAH metabolites measured in the bile. Fish also had increased levels of CYP1A protein and EROD activity in liver tissue. These results were all dose dependent, and levels of PAHs, CYP1A protein, and EROD activity all correlate significantly with one another (*p* < 0.001, Pearson’s correlation). Eelpout were also exposed to the bunker oil via an i.p. injection, which resulted in a significant increase in DNA adducts in hepatic tissue, verifying our results from the field samples. These effects have been seen previously in fish following an oil spill (Lee and Anderson 2005).

A comparison of our measurements of DNA adducts in the field samples and laboratory-treated fish revealed an interesting result. Even though the increase in DNA adducts in liver tissue is obvious after i.p. oil exposure, it could be observed that the control fish, collected at a clean reference site (Grundsund, 65 km north of Göteborg harbor) and not exposed to laboratory conditions, had an adduct level around 2.6 nmol/mol normal nucleotides, a level that suggests previous exposure to DNA adduct forming xenobiotics (Aas et al. 2003). Furthermore, when analyzing the DNA adduct fingerprint pattern in the autoradiogram from the control fish, sampled 3 months after the spill at a location 65 km to the north of the spill, an interesting observation appear. Comparison of the fingerprints suggest strong similarities in DNA adduct-forming substances between the feral control group of fish (control) and the oil-i.p.-injected-exposed fish, thereby suggesting similar exposure and a long-distance transport of substances in the oil spill. Previous studies has observed that corresponding types of DNA adduct patterns can be found over widespread areas, indicating that pollutants are able to spread and cause similar DNA damage over long distances (Ericson et al. 1998; Ericson et al. 1999).

In addition to further verifying effects of PAH-rich oil on both expression and activity of the phase I detoxification system, we show that, in situations where exposure to PAHs is very high such as those found in an oil spill, CYPA1 catalytical activity (EROD activity) can be inhibited. In vivo and in vitro studies in *Fundulus heteroclitus* (Willett et al. 2001) have demonstrated this, but propose that the inhibition of EROD activity may be due to a downregulation of CYPA1. Our results do not indicate a downregulation in CYP1A protein, as we see no decrease in levels measured using ELISA, so we propose that the effect is a result of inactivation of the catalytic activity of the CYP1A protein. A decrease in EROD activity, despite high levels of CYP1A protein, has been demonstrated in winter flounder following exposure to high concentrations of a polychlorinated biphenyl, and these effects were also found at polluted field sites (Monosson and Stegeman 1991). In either case, these results call for caution when using EROD as single biomarker of effect in similar situations as results may be misleading. Analysis of additional biomarkers, such as PAH levels in bile, may be advantageous in order to correctly interpret EROD results.
